# *Ovol2,* a zinc finger transcription factor, is dispensable for spermatogenesis in mice

**DOI:** 10.1186/s12958-019-0542-3

**Published:** 2019-11-23

**Authors:** Jin Zhang, Juan Dong, Weibing Qin, Congcong Cao, Yujiao Wen, Yunge Tang, Shuiqiao Yuan

**Affiliations:** 1 Institute of Reproductive Health, Tongji Medical College, Huazhong University of Science and Technology, Wuhan, People’s Republic of China; 2 College of Animal Science and Technology, Northwest A&F University, Yangling, People’s Republic of China; 3 NHC Key Laboratory of Male Reproduction and Genetics, Family Planning Research Institute of Guangdong Province, Guangzhou, People’s Republic of China; 4 Shenzhen Huazhong University of Science and Technology Research Institute, Shenzhen, People’s Republic of China

**Keywords:** Ovol2, Spermatogenesis, Fertility, Knockout mice

## Abstract

*Ovol2*, a mouse homolog of *Drosophila ovo*, was identified as a zinc finger transcription factor predominantly expressed in testis. However, the function of *Ovol2* in postnatal male germ cell development remains enigmatic. Here, we firstly examined the mRNA and protein levels of *Ovol2* in developing mouse testes by RT-qPCR and western blot and found that both mRNA and protein of Ovol2 are continually expressed in postnatal developing testes from postnatal day 0 (P0) testes to adult testes (P56) and exhibits its higher level at adult testis. Further testicular immuno-staining revealed that OVOL2 is highly expressed in the spermatogonia, spermatocytes and round spermatids. Interestingly, our conditional *ovol2* knockout mouse model show that loss of *ovol2* in embryonic germ cells does not affect fecundity in mice. Our data also show that *Ovol1* may have compensated for the loss of *Ovol2* functions in germ cells. Overall, our data indicate that *ovol2* is dispensable for germ cell development and spermatogenesis.

## Main text

**Fig. 1:**
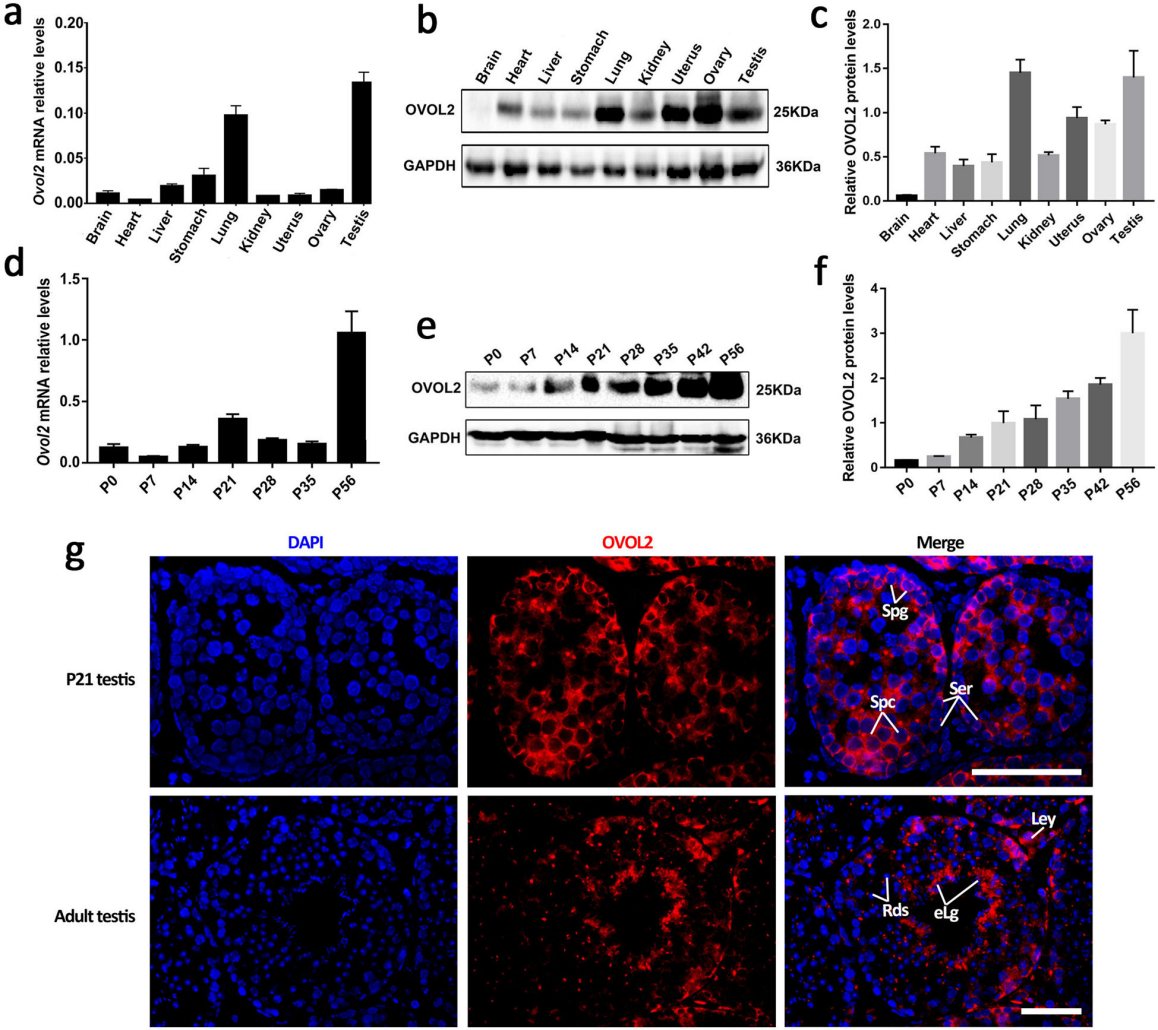
*Ovol2* is expressed in spermatogenic cells in mice. **a** RT-qPCR analyses of *Ovol2* mRNA levels in nine organs of adult mice. Data are presented as mean ± SEM, *n* = 5. **b** Western blot analyses of OVOL2 protein levels in nine organs of adult mice. GAPDH served as a loading control. **c** Quantification analyses of OVOL2 protein levels in nine organs. Data are presented as mean ± SEM, *n* = 3. **d** RT-qPCR analyses of *Ovol2* mRNA levels in developing testes. Testes at postnatal Day 0 (P0), P7, P14, P21, P28, P35, and P56 were analyzed. Data are presented as mean ± SEM, *n* = 5. **e** Western blot analyses of OVOL2 protein levels in developing testes. Testes at postnatal Day 0 (P0), P7, P14, P21, P28, P35, P42 and P56 were analyzed. GAPDH served as a loading control. **f** Quantification analyses of OVOL2 protein levels in developing testes. Data are presented as mean ± SEM, *n* = 3. **g** Immunofluorescence showing the localization of OVOL2 in P21 and adult testis sections. Note: Spg, spermatogonia; Spc, spermatocytes; Rds, round spermatids; eLg, elongated spermatids; Ser, sertoli cells; Ley, leydig cells. Scale bar = 50 μm

*Ovol2*, a mouse homolog of *Drosophila ovo*, was identified as a zinc finger transcription factor predominantly expressed in testis [1]. Previous studies revealed that *Ovol2* exhibits its functions in keratinocyte transient proliferation and differentiation [2], mouse embryonic stem cells differentiation [3] and primordial germ cell development [4]. However, the function of *Ovol2* in postnatal male germ cell development remains enigmatic. Thus, we firstly examined the mRNA and protein levels of *Ovol2* in multiple adult mouse tissues by RT-qPCR and western blot. We found that both the mRNA and protein of *Ovol2* are highly expressed in testis and lung (Fig. 1a-c). We then examined the expression levels of *Ovol2* in postnatal developing testes and found that both mRNA and protein of Ovol2 are continually expressed in postnatal developing testes from postnatal day 0 (P0) testes to adult testes (P56) and exhibits its highest level at adult testis (Fig. 1d-f). Further testicular immuno-staining revealed that OVOL2 is highly expressed in the germ cells (spermatogonia, spermatocytes and round spermatids) (Fig. 1g and Additional file 1: Figure S1A-D). Thus we hypothesized that *Ovol2* could play an important role in postnatal germ cell development and spermatogenesis.

**Fig. 2:**
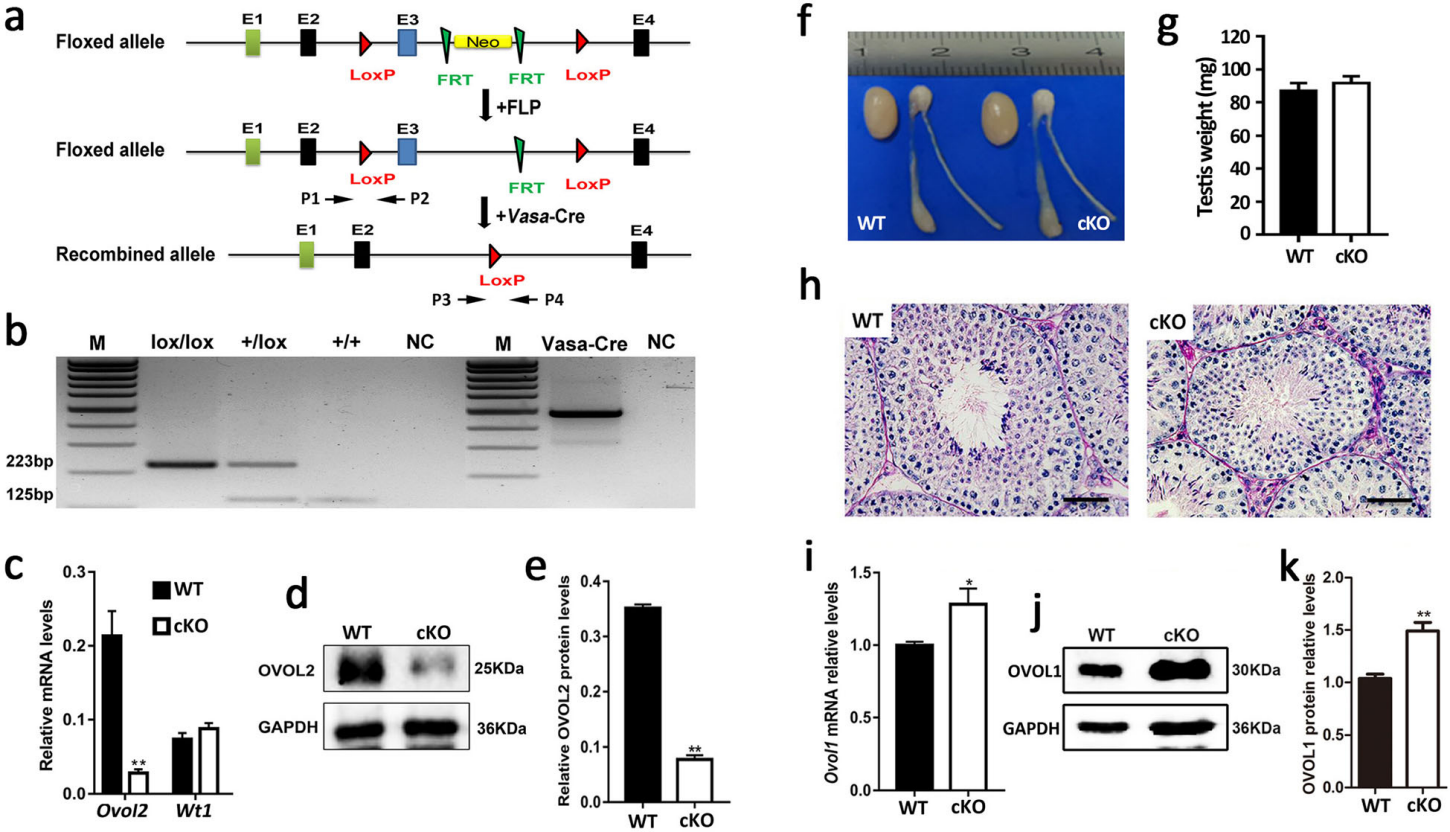
*Ovol2* is dispensable for spermatogenesis in mice. **a** Schematic illustration of the targeting strategy for generating a complete inactivation of *Ovol2* in mouse testes. Mice containing the floxed allele were crossed with *Vasa-Cre* mouse lines to generate male germ cell-specific deletion of *Ovol2* (*Vasa-Cre* was activated at embryonic day 15.5, E15.5). P1/2 indicate the position of primers used for detection of floxed and WT alleles. P3/4 indicate the position of primers used for detection of delete allele after Cre recombination. **b** Representative PCR genotyping results showing that the floxed (lox) and the WT (+) alleles can be detected as larger (223 bp) and a shorter (125 bp) bands, respectively. The last two right lanes are the Vasa-Cre transgene detection. M, marker; NC, negative control. **c** RT-qPCR analyses of *Ovol2* mRNA levels in WT and cKO (*Vasa-cre; Ovol2*^*floxflox*^) adult testes. *Wt1* gene was used as RNA quality control. Data are presented as mean ± SEM, *n* = 6. **P < 0.01 by student *t*-test. **d** Representative Western blot analysis of OVOL2 protein levels in WT and cKO (*Vasa-cre; Ovol2*^*floxflox*^) adult testes. GAPDH served as a loading control. **e** Quantification analysis of OVOL2 protein levels in WT and cKO adult testes. Data are presented as mean ± SEM, *n* = 6. *P < 0.01 by student *t-*test. **f** Gross morphology of the testis and the epididymis in WT and cKO adult mice. **g** Histogram showing the testis weights in WT and cKO adult mice. Data are presented as mean ± SEM, *n* = 6. **h** Periodic acid-Schiff (PAS) staining showing the histology of testes from adult WT and cKO mice. Scale bar = 50 μm. **i** RT-qPCR assays showing elevated *Ovol1* mRNA levels in cKO adult testes. Data are presented as mean ± SEM, *n* = 6. *P < 0.05 by student *t-*test. **j** Representative Western blot analysis of OVOL1 protein levels in WT and cKO adult testes. GAPDH served as a loading control. **k** Quantification analysis of OVOL1 protein levels in WT and cKO adult testes. Data are presented as mean ± SEM, *n* = 6. *P < 0.05 by student *t-*test

Due to *Ovol2* conventional mutant mice displayed an embryonic mortality [4], we tried to specific knockout of *Ovol2* in mouse germ cells to determine the physiological roles of *Ovol2* in germ cell development. We then generated *Ovol2* conditional knockout mouse model by crossing *Ovol2*-floxed mice with *Vasa-cre* (Cre was specifically activated at embryonic day 15.5) to inactive *Ovol2* gene in testicular germ cells (Fig. 2a). The genotype of *Ovol2* germ cell-specific knockout mice (*Vasa*-Cre; *Ovol2*^*flox/flox*^, hereafter called Vasa*-*cKO) was confirmed by PCR-based genotyping analyses (Fig. 2b). In addition to genotyping analyses, both mRNA and proteins of *Ovol2* were appeared to be significantly reduced in Vasa-cKO testes compared with that of WT controls by RT-qPCR, Western blot and Immunofluorescence analyses (Fig. 2c-e and Additional file 1: Figure S1E). Therefore, these data suggest that *Ovol2* was specifically inactivated in testes efficiently.

To investigate the fertility of Vasa*-*cKO mice, we bred the Vasa*-*cKO males with fertility-proven WT females for at least 5 months. Unexpectedly, Vasa*-*cKO breeding pairs can produce comparable litter size to WT breeding pairs (data not shown), which indicated that Vasa*-*cKO male mice are completely fertile. Consistent with this fertile phenotype, testis gross morphology and weights are comparable between Vasa-cKO and WT control mice (Fig. 2f-g). Histological analyses further revealed that Vasa*-*cKO testes display normal spermatogenesis (Fig. 2h). To further confirm the Vasa-Cre recombined deletion efficiency in DNA level, we detected the delete allele of *Ovol2* in the offspring derived from Vasa-cKO male breeding pairs by PCR-based DNA analyses. As we expected, all of pups are contained *Ovol2* delete allele (Additional file 1: Figure S2). Together, these data indicate that *Ovol2* is not essential for spermatogenesis and male germ cell development in mice despite its high expression in testis.

*Ovol1*, another *Drosophila ovo* mouse homologue, was confirmed to express in overlapping tissues with *Ovol2*, such as skin, kidney and testis. Ablation of *Ovol1* in mice led to abnormal male germ cell development and male infertility, and *Ovol1* is essential for spermatogenesis [5]. Thus, we analyzed the expression levels of *Ovol1* in adult WT and Vasa*-*cKO testes by RT-qPCR. Interestingly, both *Ovol1* mRNA and protein levels were significantly increased in Vasa*-*cKO testes compared with those of WT testes (Fig. 2i-k). Therefore, these data suggest that *Ovol1* may have compensated for the loss of *Ovol2* functions in germ cells, which leads to normal phenotype in Vasa-Cre;*Ovol2*^*lox/lox*^ mice. However, it is worthwhile pointing out that other transcription factors need to be elucidated in future, which may contribute to the compensation of OVOL2 loss-of -function in male germ cells. Overall, in this study, we report that *Ovol2* is dispensable for testicular germ cell development and spermatogenesis in mice, and provide a molecular therapeutic clue for human male infertility caused by genetic mutation.

## Conclusion

Both mRNA and protein of *Ovol2* are continually expressed in postnatal developing testes from postnatal day 0 (P0) testes to adult testes (P56) and exhibits its highest level at adult testis. *Ovol2* is dispensable for testicular germ cell development and spermatogenesis in mice. *Ovol1* may have compensated for the loss of *Ovol2* functions in germ cells, which leads to normal phenotype in *Ovol2* conditional mutation mice.

## Supplementary information

**Additional file 1: Figure S1:** The localization of OVOL2 in mouse testicular sections was revealed by immunofluorescence. **(A)** Co-­-immunofluorescent staining for OVOL2 and WT1 (a Sertoli cell marker) antibodies on P21 WT testicular section showing OVOL2 expressed in Sertoli cells. Nuclei were stained with DAPI. **(B)** Co-­-immunofluorescent staining for OVOL2 and γ-­-H2A.X antibodies on P21 testicular section showing OVOL2 expressed in pachytene spermatocytes. Nuclei were stained with DAPI. **(C)** Co-­- immunofluorescent staining for OVOL2 and γ-­-H2A.X antibodies on P56 testicular section showing OVOL2 expressed in round spermatids. Nuclei were stained with DAPI. **(D)** Co-­-immunofluorescent staining for OVOL2 and γ-­-H2A.X antibodies on P56 testicular section showing OVOL2 expressed in elongating spermatids. Nuclei were stained with DAPI. (**E**) Co-­-immunofluorescent staining for OVOL2 and γ-­-H2A.X antibodies on WT and Vasa-­-cKO (Vasa-­-cre; *Ovol2lox/lox*) testis sections at adulthood. Nuclei were stained with DAPI. Arrowheads indicate Sertoli cell; Arrows indicate Leydig cells. Scale bar bar = 50 μm. **Figure S2.** PCR based genotyping of the offspring derived from the Vasa-­-cKO (Vasa-­- cre; Ovol2lox/del) male breeding pairs. All pups are contains delete allele.
