# Supplementary material for: Ovol2, a zinc finger transcription factor, is dispensable for spermatogenesis in mice
Source: Reprod Biol Endocrinol. 2019 Nov 23;17:98. doi: 10.1186/s12958-019-0542-3 (PMC6875160; doi:10.1186/s12958-019-0542-3)
Supplement: Supplementary file 1 — Additional file 1: Figure S1: The localization of OVOL2 in mouse testicular sections was revealed by immunofluorescence. (A) Co-­-immunofluorescent staining for OVOL2 and WT1 (a Sertoli cell marker) antibodies on P21 WT testicular section showing OVOL2 expressed in Sertoli cells. Nuclei were stained with DAPI. (B) Co-­-immunofluorescent staining for OVOL2 and γ-­-H2A.X antibodies on P21 testicular section showing OVOL2 expressed in pachytene spermatocytes. Nuclei were stained with DAPI. (C) Co-­- immunofluorescent staining for OVOL2 and γ-­-H2A.X antibodies on P56 testicular section showing OVOL2 expressed in round spermatids. Nuclei were stained with DAPI. (D) Co-­-immunofluorescent staining for OVOL2 and γ-­-H2A.X antibodies on P56 testicular section showing OVOL2 expressed in elongating spermatids. Nuclei were stained with DAPI. (E) Co-­-immunofluorescent staining for OVOL2 and γ-­-H2A.X antibodies on WT and Vasa-­-cKO (Vasa-­-cre; Ovol2lox/lox) testis sections at adulthood. Nuclei were stained with DAPI. Arrowheads indicate Sertoli cell; Arrows indicate Leydig cells. Scale bar bar = 50 μm. Figure S2. PCR based genotyping of the offspring derived from the Vasa-­-cKO (Vasa-­- cre; Ovol2lox/del) male breeding pairs. All pups are contains delete allele. [file 12958_2019_542_MOESM1_ESM.pdf]

Supplementary Figures

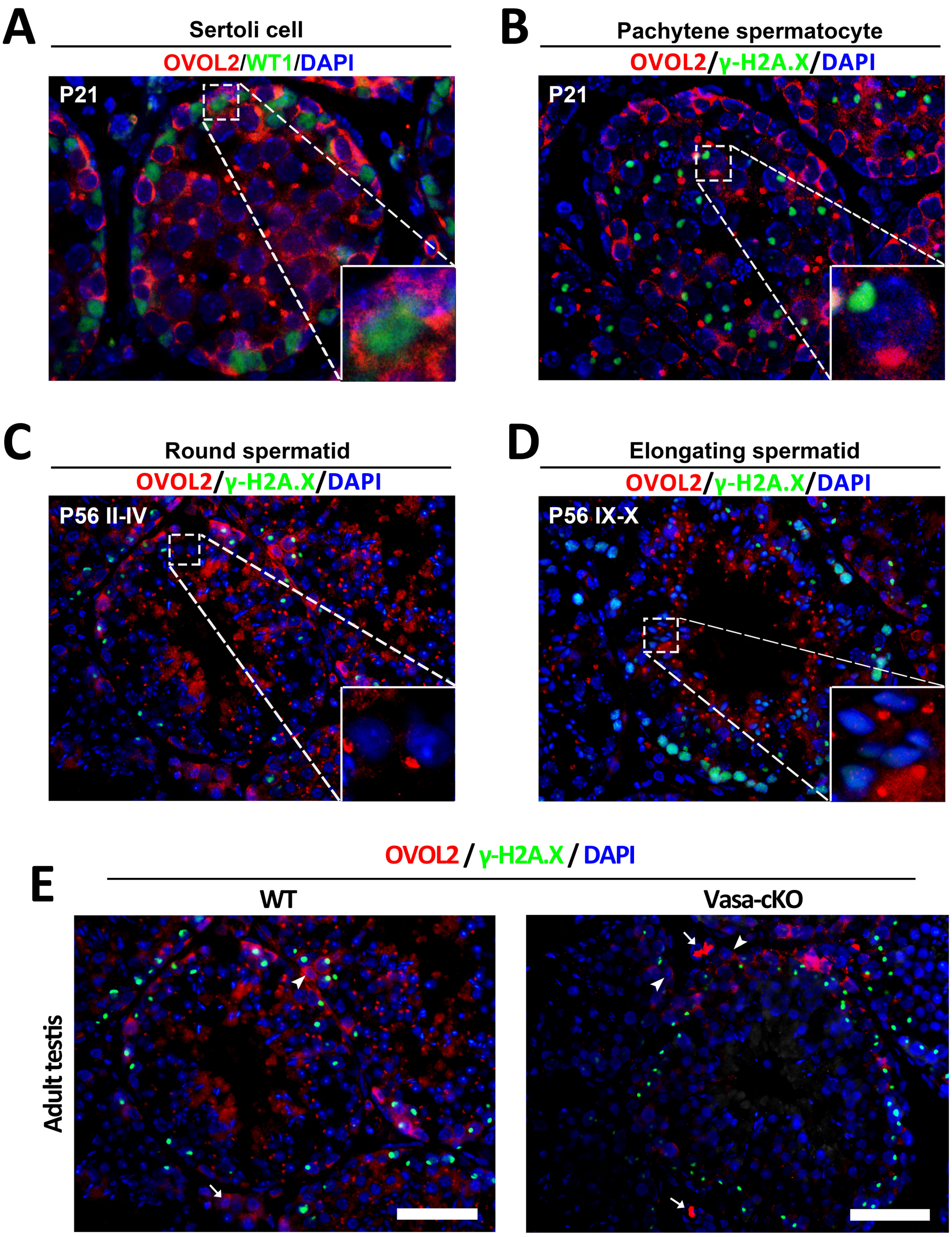

Figure S1. The localization of OVOL2 in mouse testicular sections was revealed by immunofluorescence. **(A)** Co-immunofluorescent staining for OVOL2 and WT1 (a Sertoli cell marker) antibodies on P21 WT testicular section showing OVOL2 expressed in Sertoli cells. Nuclei were stained with DAPI. **(B)** Co-immunofluorescent staining for OVOL2 and γ-H2A.X antibodies on P21 testicular section showing OVOL2 expressed in pachytene spermatocytes. Nuclei were stained with DAPI. **(C)** Co-

immunofluorescent staining for OVOL2 and γ-H2A.X antibodies on P56 testicular section showing OVOL2 expressed in round spermatids. Nuclei were stained with DAPI. **(D)** Co-immunofluorescent staining for OVOL2 and γ-H2A.X antibodies on P56 testicular section showing OVOL2 expressed in elongating spermatids. Nuclei were stained with DAPI. (**E**) Co-immunofluorescent staining for OVOL2 and γ-H2A.X antibodies on WT and Vasa-cKO (Vasa-cre; *Ovol2$^{lox/lox}$*) testis sections at adulthood. Nuclei were stained with DAPI. Arrowheads indicate Sertoli cell; Arrows indicate Leydig cells. Scale bar bar=50µm.

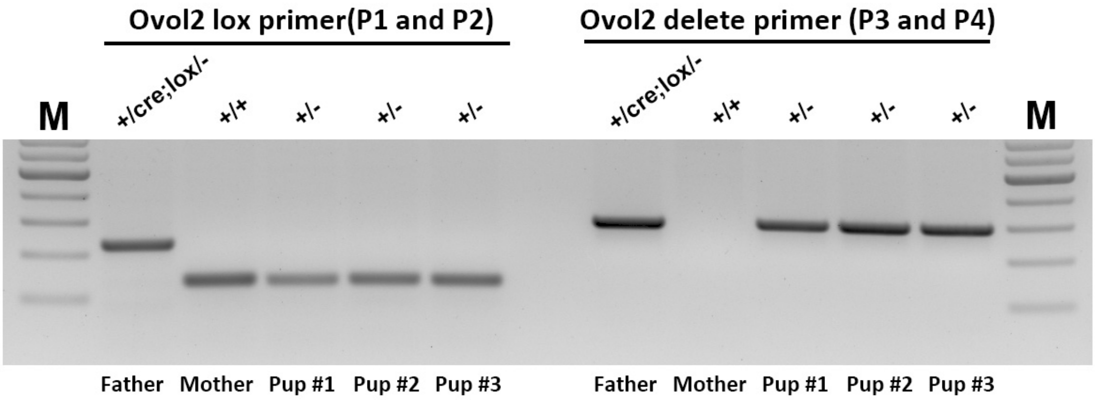

Figure S2. PCR based genotyping of the offspring derived from the Vasa-cKO (Vasa-cre; Ovol2$^{lox/del}$) male breeding pairs. All pups are contains delete allele.
